# Influence of Manure as a Complex Mixture on Soil Sorption of Pharmaceuticals—Studies with Selected Chemical Components of Manure

**DOI:** 10.3390/ijerph20126154

**Published:** 2023-06-16

**Authors:** Sören Thiele-Bruhn, Wei Zhang

**Affiliations:** 1 Department of Soil Science, Trier University, Behringstraße 21, 54296 Trier, Germany; weizhang@ctbu.edu.cn; 2 Department of Land Resources Management, Chongqing Technology and Business University, Xuefu Avenue 19, Chongqing 400067, China

**Keywords:** atenolol, caffeine, sulfadiazine, dissolved organic matter, sewage, specific sorption, sorption competition

## Abstract

Pharmaceutically active compounds (PhACs) enter soil with organic waste materials such as manure. Such complex substrates differently affect PhACs’ soil sorption. For the first time, batch experiments were conducted using five selected chemicals as model constituents to elucidate the effects. Urea, phosphate (KH_2_PO_4_), acetic acid, phenol and nonadecanoic acid (C:19) altered the sorption strength and/or nonlinearity of sulfadiazine, caffeine, and atenolol in an arable Cambisol topsoil. The nonlinear Freundlich model best described sorption. Overall, the PhACs’ Freundlich coefficients (sorption strength) increased in the sequence urea < phosphate < phenol < C:19 < acetic acid, while the Freundlich exponents largely decreased, indicating increasing sorption specificity. The effects on sulfadiazine and caffeine were rather similar, but in many cases different from atenolol. Phosphate mobilized sulfadiazine and caffeine and urea mobilized sulfadiazine, which was explained by sorption competition resulting from specific preference of similar sorption sites. Soil sorbed phenol strongly increased the sorption of all three PhACs; phenolic functional groups are preferred sorption sites of PhACs in soil. The large increase in sorption of all PhACs by acetic acid was attributed to a loosening of the soil organic matter and thus the creation of additional sorption sites. The effect of C:19 fatty acid, however, was inconsistent. These results help to better understand the sorption of PhACs in soil–manure mixtures.

## 1. Introduction

Pharmaceutical active compounds (PhACs) such as antibiotics, analgesics, and beta blockers, to name a few, are highly potent chemicals [1]. Since the early work on PhACs in the soil environment around the turn of the millennium [2,3,4,5,6], it has become clear that PhACs can no longer be called emerging contaminants, but residual contamination of the soil environment with PhACs is a ubiquitous problem [7,8,9]. Residual concentrations of PhACs in soil are typically in the range of a few to several hundred micrograms per kilogram of soil [9,10,11,12], and hence in the same range as those of pesticides. Similar to “pesticide”, PhAC is a collective term for many different classes of small- to medium-sized molecular compounds. However, one of the main differences is that PhACs, except for a few agents against bacterial pathogens in plants, are not intentionally released into the soil environment. Consequently, the adverse effects of PhACs are unintended but can be significant, such as the impact on soil antibiotic resistance [13,14], which, in the sense of the One Health approach, also endangers human health [15,16]. In addition, the vast majority of PhAC inputs come from livestock manure, as well as sewage (biosolids) and wastewater from humans (for simplicity all these substrates are referred to as “slurry” in the following) [17,18,19,20]. This is because PhACs are predominantly and rapidly excreted from the treated body [21,22] and the excreta are subsequently used for fertilization or irrigation of agricultural soils. Concentrations in slurry range from a few up to several thousand micrograms per kilogram [11], with mixed multi-component contaminations occurring in most cases [23].

Manure and similar substrates are highly complex mixtures of diverse constituents. These constituents range (a) in size from particulate tissues, e.g., residual feed and bedding material, to truly dissolved single molecules, (b) in chemical identity from components such as salts to organic macromolecules, and (c) from highly polar to very non-polar compounds, while (d) the overall composition varies depending on the organism type, age, diet, and storage conditions of the slurry [24,25,26,27]. Adding slurry to soil alters the composition of the soil organic matter (SOM), resulting in enrichment of manure-specific compound classes [28,29] and a significant increase in the amount of dissolved organic matter (DOM) [30]. This result greatly influences the fate and especially the sorption of PhACs in the soil, the latter being one of the most important soil properties to mitigate environmental pollution.

The different interactions between slurry of different compositions, PhACs, and soil is reflected in contrasting reports in the literature on the effects of slurry on the soil sorption of PhACs, ranging from increased immobilization [31,32,33] to increased mobilization and transport [34,35,36]. These results indicate that the overall effect depends largely on the individual combination of the ternary mixture of soil—PhACs—slurry and especially on the composition of the slurry. This has been investigated in studies focusing on the soil sorption of PhACs in the presence of slurry from different sources and slurry fractions such as dissolved organic matter (DOM) and polarity fractions (e.g., [37,38,39,40]). In contrast, the impact of different chemical components of manure has, to the best of our knowledge, not yet been investigated.

Accordingly, it was the aim of this study to investigate for the first time the effect of selected pure chemicals, representing relevant constituents of manure [41,42,43,44,45], on the soil sorption of PhACs. This was done by using urea and potassium dihydrogen phosphate (KH_2_PO_4_) as major nutrient components, acetic acid and phenol as representatives of volatile organic compounds, and nonadecanoic acid (C:19) as a non-polar organic constituent of manure. The antibiotic sulfadiazine, the beta-blocker atenolol, and the psychotropic drug caffeine were tested as PhACs, which had been previously shown to differently interact with manure fractions [46]. Thus, this study followed up on previous research to further elucidate sorption in ternary soil–fertilizer–PhAC mixtures [46]. It was hypothesized that the selected model compounds affect the sorption of the PhACs differently.

## 2. Materials and Methods

A soil sample representing a typical arable topsoil in a temperate region was used for the sorption experiments. Briefly, the topsoil of a loamy sand, Haplic Cambisol was sampled from a 1.5 ha field site near Ferschweiler in the region of Trier (N 49°51′77″; E 6°49′26″) from a depth of 0–15 cm. The composite sample (five individual corings) was taken in early spring before the start of the growing season of the winter wheat crop (*Triticum aestivum* L.). The soil sample was also used in our previous study [46] (there, it was sample II of five soil samples). It was selected because it showed good sorption of the tested PhACs and a clear effect of manure DOM on the sorption. Most importantly, the acidic pH of the soil ensured that pH changes due to the addition of the selected model compounds would not alter the speciation of the three PhACs tested to such an extent that the pH effect alone would explain the altered sorption. 

Soil pH and electrical conductivity (EC) were determined by a pH meter (electrode SenTix 21, WTW, Weilheim, Germany) and conductivity meter (Cond 340i, WTW, Weilheim, Germany), respectively, in 0.01 M CaCl_2_ at a soil to solution ratio of 1:2.5. The soil organic carbon (SOC) and nitrogen content of the soil were measured by elemental analysis (EA 3000, Hekatech, Wegberg, Germany). The concentrations of the amorphous (oxalate-extractable) iron oxides (Fe_o_) were determined by the modified method of Schwertmann [47] and using graphite furnace atomic absorption spectrometry (ContrAA 700 High Resolution Continuum Source, Varian, Palo Alto, CA, USA). The investigated soil properties were as follows: pH 4.98; EC (mS·cm^−1^) 2.43; SOC (mg·g^−1^) 11.57; C/N ratio 11.25; Fe_o_ (%) 0.18; CEC (mmol_c_·kg^−1^) 37.18; clay (%) 6.0.

Analytical grade standard chemicals of sulfadiazine, caffeine, and atenolol were purchased from Dr. Ehrenstorfer GmbH (Augsburg, Germany). Selected physicochemical properties of the tested PhACs are listed in Table 1. Methanolic stock solutions of the PhACs were prepared at a concentration of 1 mg·mL^−1^ and stored at 4 °C in the dark. 

In addition, five pure model compounds, i.e., urea, monopotassium phosphate (phosphate), acetic acid, phenol, and nonadecanoic acid (C:19), were investigated. These model compounds resemble different components of manure. The chemicals were obtained from Merck (Darmstadt, Germany), C:19 was obtained from Supelco (Darmstadt, Germany), and all were of analytical grade or higher purity.

To investigate the sorption of the three PhACs in the presence of manure constituents, the aforementioned five pure model compounds were added to soil at a spiking level of 5 mmol·g^−1^. The spiking level was roughly estimated based on the composition of manure [24,25,26,27] and the typical addition rates of manure to soil [23]. However, in this study, all model compounds were tested at the same concentration to obtain comparable results, even though their concentrations in manure vary. All samples were combined with one of the three PhACs added at two different spiking concentrations, i.e., 20 and 70 μg·g^−1^, covering typical residual concentrations in field soils [23]. Additionally, samples without PhACs (0 μg·g^−1^) were investigated. Furthermore, control samples without addition of the model compounds (spiking levels 0, 1, 20, 50, 70, and 100 μg·g^−1^ [46]) were investigated. All samples were prepared in triplicate.

The sorption of sulfadiazine, atenolol, and caffeine to the soil sample in the presence and absence of pure model compounds was investigated according to OECD guideline 106 [49]. For each sample, 5 g of soil was suspended in 0.01 M CaCl_2_ (soil to liquid ratio = 1:2.5 *w*/*w*) and equilibrated for 12 h to fully remoisten the soil. Then, the model compounds and PhACs were added, and the samples were agitated on an end-over-end shaker at 22 °C at 15 rpm for 72 h. The equilibrium time of 72 h was determined in a preliminary kinetic experiment [46]. Afterwards, samples were immediately centrifuged at 2000× *g* for 30 min. In order to analyze the PhACs, the supernatants were solid-phase extracted using an HR-X cartridge (Macherey–Nagel, Düren, Germany) preconditioned with 6 mL of methanol followed by 6 mL of HPLC-grade water. After the supernatant was passed through the cartridge, it was rinsed with 6 mL of a methanol–water mixture (2:8 *v*/*v*) and subsequently dried in a nitrogen gas stream for 30 min. Finally, the target PhACs were eluted from the cartridge using 6 mL methanol. The eluted volume was evaporated to about 0.5 mL in a rotary evaporator (Rotavapor R-114, Flawil, Switzerland) and redissolved in 1.0 mL methanol. Samples were spiked with 0.5 µg sulfadimidine as internal standard for quantification and transferred to amber LC autosampler vials. Using this method, sulfadiazine, atenolol, and caffeine were determined at the aforementioned spiking concentrations with recovery rates of 84.03%, 79.49%, and 90.15%, respectively. The degradation of the three PhACs over 72 h was negligible in preliminary tests [46].

Analytical determination of the pharmaceuticals was done by using LC-ESI-MS/MS. Chromatographic separation was achieved with a Hypersil Gold C18 HPLC column (50 × 2.1 mm, 3.0 µm, Thermo Electron, Karlsruhe, Germany) as stationary phase and HPLC water with 0.1% formic acid (*v*/*v*; eluent A) and methanol with 0.1% formic acid (*v*/*v*; eluent B) as mobile phases. A gradient program was used to deliver the mobile phases at a flow rate of 0.2 mL min^−1^ with 98% of eluent A as initial condition, linearly increased within 10 min to 100% of eluent B, 4 min isocratic elution, and 1 min linear gradient back to 98% of eluent A, the latter held for 2 min to equilibrate the column. The sample injection volume was 10 µL. The chromatographic system consisted of a Shimadzu LC-20 HPLC (Shimadzu, Duisburg, Germany) coupled to an API 3200 LC–ESI–MS/MS (Applied Biosystems/MDS Sciex Instruments, Toronto, ON, Canada) operated in positive ion mode. The settings of the ion-source were as follows: ion spray voltage 5000 V; source temperature 400 °C; collision gas 7 psi; curtain gas 25 psi. The Analyst 1.4.2 software (Applied Biosystems/MDS Sciex Instruments, Toronto, ON, Canada) was used for peak integration and data assessment. The evaluation of chromatograms was done as reported by Ngigi, et al. [50]. The analytical method yielded limits of detection of 5 µg·L^−1^ and limits of quantification (LOQ) of 10 µg·L^−1^. All the data reported in this study were higher than the LOQ.

The sorption data were described using the Freundlich, Langmuir, and linear isotherm models to identify the best fitting model. The three models were selected based on the results of numerous previous studies showing that these models are best suited to describe soil sorption of polar PhACs (e.g., [46,51,52,53]). Of the three equations selected, the Freundlich model achieved the best fit to the data. Therefore, only the results of the Freundlich model (Equation (1)) are presented and discussed.

*q_e_* = *K_f_* × *c_e_^n^* (1)

where *q_e_* (µmol·g^−1^) is the equilibrium adsorbed concentration and *c_e_* (µmol·mL^−1^) is the equilibrium solution concentration of the pharmaceutical. The parameters *K*_f_ (µmol^(1 − n)^·mL^n^·g^−1^) and *n* denote the Freundlich sorption coefficient and the Freundlich exponent as measure of the isotherm’s nonlinearity. In order to better compare the sorption coefficients of isotherms with different nonlinearity, the linear sorption coefficient (*K_d_*) was additionally calculated from *K_f_* using Equation (2) [32].

*K_d_* = *K_f_* × *c_e_^n^*^−1^. (2)

For this purpose, *K_d_* (mL·g^−1^) was calculated in this study based on an equilibrium concentration (*c_e_*) of 10 µmol·mL^−1^. From the *K_d_*, the *K_OC_* was calculated according to Equation (3).

*K_OC_* = *K_d_*/SOC (%) × 100. (3)

Curve fitting by nonlinear regression was done using the SigmaPlot 14 software (Systat GmbH, Frankfurt/Main, Germany). 

To determine differences between isotherms, graphs were described by their confidence intervals [3]. The half-width *A* of a confidence interval at a solution concentration *c_W_* (mg L^−1^) and a probability of error of α were calculated (Equation (4)).

(4) $$A=SE\times t_{N-2,\alpha /2}\times \sqrt{\frac{1}{N}+\frac{{\left(lgc_{w}-lgc_{wq}\right)}^{2}}{\left(\sum lgc_{wi}{}^{2}\right)-{\left(\sum lgc_{wi}\right)}^{2}\times 1/N}}$$

*SE* was the standard error of the regression, *t* the *t*-quantile, *N* the number of observations, *c_wi_* the measured solution concentration of *i* = 1 – *N*, and *c_wq_* the mean solution concentration. Differences between isotherms were significant when the difference of two isotherms at a given solution concentration *c_W_* exceeded the sum of half the confidence intervals. The confidence interval was (Equation (5)):

(5) $$\left[lgK_{f}+n\times lgc_{w}-A;\;lgK_{f}+n\times lgc_{w}+A\right].$$

## 3. Results

The soil pH and SOC content as relevant soil parameters were changed in the presence of the model substances as shown in Table 2. Adding the organic chemicals urea, acetic acid, and phenol slightly increased the total SOC content, while the C:19 fatty acid with the highest relative C content in the molecular structure (Table 1) increased the SOC content by almost 10%. The effect on soil pH was by far the greatest in the presence of acetic acid with a decrease from pH 4.98 to 2.56, whereas all other compounds decreased pH by 0.6 pH units or less within an equilibrium time of 72 h (Table 2). 

**Figure 1:**
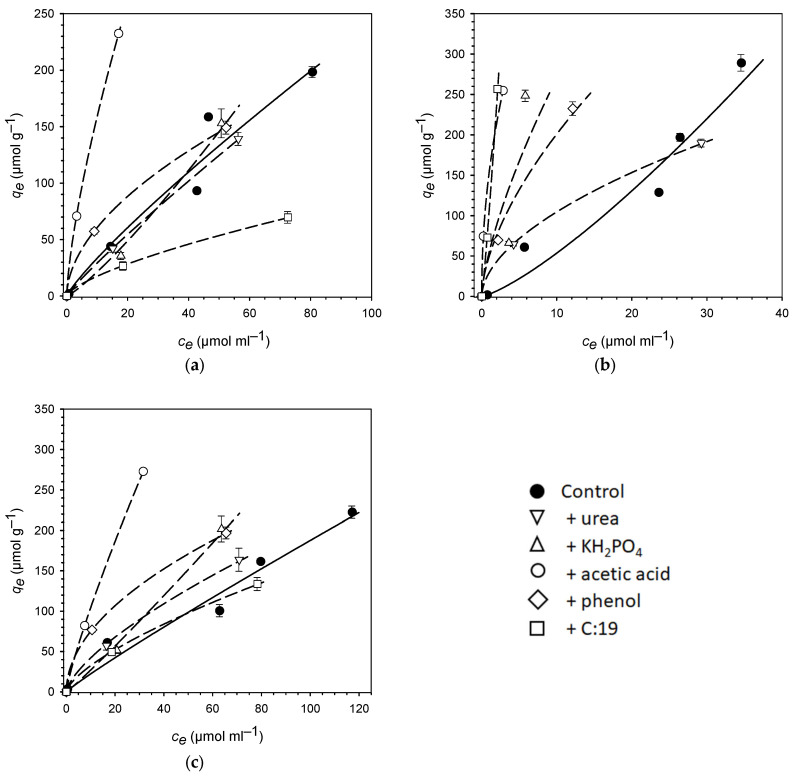
Sorption of PhACs ((**a**) sulfadiazine, (**b**) atenolol, (**c**) caffeine) to soil (topsoil of a Haplic Cambisol) in the absence (Control) or presence of different model compounds representing chemical constituents of manure, added to soil at 5 mmol·g^−1^. Dissolved and adsorbed equilibrium concentrations are displayed (mean values with standard deviation as error bars) and were fitted using the Freundlich isotherm model (Equation (1); lines). Note the different scaling of the axes in the sub-figures. Data for the control were taken from [46].

The slightly nonlinear sorption of the three PhACs in soil was well described by the Freundlich sorption isotherm model (Figure 1). It should be noted that the sorption of the PhACs to the control samples (without the addition of manure model compounds) was investigated in a previous study by testing six concentration levels [46]. Based on these results and the goodness of fit of the model, it was concluded that the Freundlich model was also best suited to describe the smaller data sets, with only three concentration levels obtained in this study. The goodness of the model fit was reflected in the statistical parameters in question, e.g., a coefficient of determination (*R*^2^) of 0.92 and higher and a standard error (*SE*) of 4.28 and less (Table 3). However, the fit by the model was significantly weaker for the combination of atenolol and KH_2_PO_4_, with *R*^2^ of 0.48 and *SE* of 24.3.

Soil sorption of all three PhACs was largely affected by the addition of the different manure model compounds. Overall, the effects were highly variable, ranging from decreased to increased mobility for sulfadiazine, while mobilization was observed in all cases for atenolol and mobilization or no significant effect for caffeine (derived from confidence intervals, see Equations (4) and (5)). On average of all three PhACs, the sorption coefficients (*K_f_* and *K_d_*) increased in the presence of the model compounds in the sequence urea < phosphate < phenol < C:19 < acetic acid. However, clear differences were observed between the individual combinations of model substances and PhACs. Similarly, the Freundlich exponents (*n*) as a measure of the sorption nonlinearity were altered differently in the different combinations with the model substances. In most cases, the exponents were reduced, and the nonlinearity of the isotherms increased. The reduction in the Freundlich exponent *n* by the model substances was as follows: phosphate reduced *n* the least and even increased *n* for the sorption of sulfadiazine and caffeine, while *n* decreased more and more with the addition of C:19, urea, acetic acid, and phenol (Table 3). In general, the effects on the sorption of sulfadiazine and caffeine were rather similar, while the effects on the sorption of atenolol were very different in many cases.

Phosphate decreased the Freundlich sorption coefficient (*K_f_*) of sulfadiazine and caffeine (and *K_d_* of sulfadiazine). At the same time, and only in these two cases, the sorption exponents (*n*) were increased, reaching values >1. The antibiotic SDZ was also slightly mobilized in the presence of urea and C:19, as was indicated by reduced sorption coefficients. In comparison, caffeine was not mobilized by urea and C:19. Instead, sorption coefficients increased, although the enhanced immobilization was weakest compared to the other model substances. In contrast, the sorption coefficient of atenolol strongly increased by a factor of 38.4 in the presence of the C:19 fatty acid, while the Freundlich exponent slightly declined but remained at a value >1. Both phenol and acetic acid strongly increased the sorption coefficients of all three PhACs; *K_d_* increased by factors of 1.8 to 3.6 for phenol and 4.6 to 8.5 for acetic acid. 

Apparently, the SOC content significantly (*p* < 0.05) explained the soil sorption of atenolol with a Pearson correlation coefficient (*r*) of 0.942, while this was not the case for sulfadiazine and caffeine (Table 4). However, the correlation of *K_d_* values of atenolol with SOC was very much governed by the *K_d_* in the presence of C:19, since C:19 provided the most additional SOC to the soil sample (Table 2) and resulted in by far the strongest sorption of atenolol (Figure 1b; Table 3). Removing this extreme data point from the correlations resulted in a non-significant relationship between the SOC content and soil sorption, even for atenolol (Table 4). Accordingly, normalization of *K_d_* to *K_OC_* (Table 3) did not result in a closer relationship between the sorption coefficient and the SOC content. Contrary to theoretical expectations, normalizing the *K_OC_* to the SOC content of the soil sample did not result in an alignment of the sorption coefficients (Table 3).

The pH was negatively correlated (*p* < 0.05) with the sorption coefficients of sulfadiazine and caffeine in the soils amended with the different model compounds (Table 4). No such correlation, however, was found for atenolol. When the values for *K_d_* in the presence of acetic acid, which had lowered the pH of the samples the most, were removed, all correlations became non-significant. Furthermore, the speciation of the three PhACs remained almost unaffected despite the pH changes. Both atenolol and caffeine occurred solely as cationic species throughout the range from pH 2.56 to 4.98. The distribution of sulfadiazine between cationic, neutral, and anionic species started from pH 4.98 with 0.04%, 97.03%, and 2.93%, respectively, reached a maximum of neutral species of 99.07% at pH 4.40 and decreased substantially to 90.71% neutral species but 9.28% cationic species at the lowest pH of 2.56.

## 4. Discussion

The mostly minor effects of the five model compounds on the soil pH and on the SOC content of the soil sample (Table 2) were expected. The very slight pH decrease despite the presence of alkaline urea indicated that some hydrolyzation to ammonium and further oxidation to nitrate had occurred within the equilibrium time of 72 h [54]. Hence, it must be expected that not only the intact urea molecule but also ammonium and nitrate ions influenced the sorption of the three PhACs. 

It is well known that SOC content and pH are soil properties that dominate the sorption of polar PhACs [55,56]. However, the weak or non-significant correlations indicated that the effects of the model compounds on the soil sorption of the PhACs were not solely due to the changes in these two soil properties (Table 4). The inability of SOC content alone to explain soil sorption of PhACs was already reported in the early work of Tolls [4] and subsequently confirmed many times (e.g., [33]). For example, in addition to SOM, mineral soil colloids are highly relevant as sorbents for polar compounds [55,57]. Furthermore, the nonlinearity of PhACs’ sorption isotherms (Figure 1) showed that soil sorption was not based on partitioning into SOM (Table 2). This was also confirmed by the increase in the nonlinearity of the sorption isotherms in the presence of the model compounds, which can be read from the decreasing Freundlich exponents (*n*). It is well known that a decline of *n* can be interpreted as an increase in sorption heterogeneity [58]. Accordingly, it is presumed that the added model compounds, when sorbed in soil, resulted in an increase in specific sorption sites for PhACs or, in some cases, competition for specific sorption sites with PhACs. It is noted that soil minerals, i.e., clay minerals and pedogenic oxides, also contribute significantly to soil sorption of PhACs [3,7]. This was not investigated in this study, but it can be assumed that the PhACs were adsorbed to both the organic and mineral matter of the soil.

Sorption competition occurred when sulfadiazine or caffeine were combined with phosphate as was previously observed [59]. Phosphate ions especially occupy the sorption sites at metal oxide minerals through ligand exchange [60], which are thus no longer available to the PhACs as likewise preferred sorption sites [52]. The increase in the Freundlich exponent *n* to values >1 indicated such sorption competition, which is particularly effective at low spiking concentrations. 

The result that only sulfadiazine was mobilized by urea, whereas caffeine and atenolol were more strongly sorbed (Table 3), indicated a very specific interaction. Sulfonamides such as sulfadiazine are known to bind to sorption sites via the amino group [55,61]. Urea and ammonium formed from it compete for the same sorption sites. The slight increase in sorption of cationic caffeine and atenolol instead may be due to the small effect of urea on soil pH. It may also indicate that atenolol with an amino group adjacent to an electronegative oxo group in the molecular structure (Table 1) does not sorb in the same way as sulfadiazine.

Similar to urea, the C:19 fatty acid also slightly mobilized sulfadiazine, while the soil sorption of caffeine was slightly and that of atenolol was strongly increased (Table 3). This was surprising given the speciation of the three PhACs; an association of the neutral sulfadiazine with the largely nonpolar fatty acid was hypothesized. Atenolol may have sorbed more strongly with the fatty acid because of its theoretically slightly lower polarity (higher *K*_OW_, Table 1). It should be noted that the effects on soil sorption can vary greatly for each specific combination of PhAC and organic component [62]. Further research on sorption mechanisms is needed to elucidate these results.

Phenol strongly increased the sorption coefficients of all three PhACs and had even the strongest effect on the sorption heterogeneity of sulfadiazine and caffeine (Figure 1; Table 3). The sorption of phenol itself is largely governed by the association with organic matter and is rather weak [63], with reported soil sorption coefficients being a factor of ten smaller than the soil sorption coefficients determined for the three PhACs in this study. As a result, phenol did not compete with PhACs for sorption sites but instead provided new and more heterogeneous sorption sites. Accordingly, naturally occurring phenolic compounds and phenolic functional groups contribute substantially to the sorption of PhACs in soil [61,64].

A very strong increase in sorption coefficients of the PhACs was found in the presence of acetic acid (Figure 1). In contrast, the effect on sorption nonlinearity was rather weak for sulfadiazine and caffeine. This may indicate that acetic acid increased the sorption without altering the sorption mechanisms. This is only partly explained by the strong effect on pH (Table 2), which hardly led to a change in the speciation of the three PhACs (see results section) but impedes the sorption of the tested PhACs’ cationic species. It seems more significant that acetic acid leads to a loosening and partial dissolution of the supramolecular structure of SOM [65]. This would create and open additional, but not different, sorption sites for the PhACs. The partial dissolution of the supramolecular structure would also have led to the formation of additional dissolved organic matter (DOM). PhACs form mobile associations with DOM [34,66]. However, such associations could not be explicitly investigated in this study due to the solid phase extraction method used, which also captures DOM associates as part of the dissolved fraction. Since a decrease in dissolved fractions was even observed in the presence of acetic acid, there is no evidence of mobilization by additional DOM.

In most cases, the tested manure constituents led to an increased soil sorption of the three PhACs. This is consistent with the effect of adding manure and sewage sludge to soils. These substrates, with their high solid phase content, often result in greater immobilization of PhACs [33,67]. However, it was expected that the pure chemicals tested would act more like manure-derived DOM and have a mobilizing effect [34,66,68]. Instead, the contrasting results of this study showed that the colloidal properties of DOM result in a different effect on the sorption of PhACs compared to pure chemicals. In addition, the effects of complex mixtures of compounds, such as those found in manure-derived DOM, are very much defined by the different constituents and their proportions in the mixture. Accordingly, opposing effects of manure-derived DOM on the sorption of PhACs have also been reported [69,70].

## 5. Conclusions

Batch experiments with selected pure chemicals serving as model compounds for manure constituents successfully demonstrated the differential effects on the mobility and sorption of PhACs in soil. It became clear that it is not the effects on soil properties, i.e., organic matter content and pH, but rather the specific interactions of these individual model compounds with sorption sites in the soil that in turn create new sorption sites for PhACs or, less often found in this study, hinder the sorption of PhACs. Manure, sewage sludge and wastewater, however, are complex mixtures of myriads of different individual compounds. If the components investigated here, and possibly others, are introduced into soils in mixtures, additive, synergistic, or antagonistic interactions of these components with respect to soil sorption of PhACs must also be expected. Therefore, the effects of manure on soil sorption of PhACs or even other agrochemicals are likely to be more complex than these initial experiments to elucidate the effects of different material components of manure have been able to show. Further research is needed on this. The results of this study using dissolved model compounds differ from the findings on the predominantly mobilizing effect of manure-derived DOM on the sorption of PhACs in soil. In contrast, this study found a predominantly immobilizing effect of most of the model compounds tested. It is suggested that it is the colloidal fractions of DOM, rather than the dissolved individual chemical constituents of DOM, that are relevant to this divergent effect on the soil sorption of PhACs. In the future, this and further studies on manure components in combination with soils of different composition and properties will allow a better prediction of the complex influence of manure on the soil sorption of PhACs.

## Figures and Tables

**Table 1:** Molecular structure and chemical properties of the three selected PhACs as well as of the five chemicals used as model compounds of manure.

Compound	Molecular	CAS Number	Molar Mass	p*K*_a_		*K*_OW_ ^a^	Water Solubility
	Formula		(g·mol^−1^)	1	2		(mg·L^−1^)
Pharmaceutical active compounds (PhACs)							
Sulfadiazine	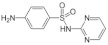	68-35-9	250.30	1.57 ^b^	6.50 ^b^	0.812 ^b^	2000
Atenolol	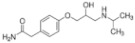	29122-68-7	266.34		9.60 ^c^	1.445 ^d^	429
Caffeine	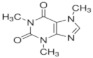	58-08-2	194.19	0.40 ^c^	10.4 ^c^	0.851 ^c^	21,600
Model compounds (manure constituents) ^e^							
Urea	CO(NH_2_)_2_	57-13-6	60.06	0.18		0.008	5.45 × 10^5^
Monopotassium phosphate	KH_2_PO_4_	7778-77-0	136.09	2.15	6.82	-	2.22 × 10^5^
Acetic acid	CH_3_COOH	64-19-7	60.05	4.76		−0.17	4.76 × 10^5^
Phenol	C_6_H_5_OH	108-95-2	94.11	9.99		28.84	8.28 × 10^4^
Nonadecanoic acid C:19	C_18_H_37_COOH	646-30-0	298.50	4.78		2.75×10^8^	0.002

^a^ *K*_OW_ of the neutral species; ^b^ data from [48]; ^c^ http://www.drugbank.ca; ^d^ http://www.toxnet.nlm.nih.gov; ^e^ US-EPA EPISuite; all internet resources accessed on 12 September 2022.

**Table 2:** Soil organic carbon content (SOC) and pH of samples without addition of model compounds (control) or equilibrated with 5 mmol·g^−1^ of five different model compounds representing relevant chemical constituents of manure.

	Control	+Urea	+KH_2_PO_4_	+Acetic Acid	+Phenol	+C:19
SOC (mg·g^−1^)	11.57	11.63	11.57	11.78	11.93	12.71
pH	4.98	4.90	4.40	2.56	4.49	4.46

**Table 3:** Freundlich model parameters (*K_f_* and *n*) and derived linear sorption coefficients (*K_d_* and *K_OC_*) for soil sorption of PhACs without (Control) or with the addition of five different model compounds.

PhAC	Isotherm Parameter	Control	+Urea	+KH_2_PO_4_	+Acetic Acid	+Phenol	+C:19
Sulfadia	*K_f_*	4.66	3.71	1.39	31.1	17.5	3.56
zine	*n*	0.86	0.90	1.19	0.71	0.54	0.69
	*R* ^2^	0.94	0.99	0.92	0.99	0.99	0.97
	*SE*	2.41	1.15	1.43	3.85	2.55	1.26
	*K_d_*	3.36	2.94	2.15	15.9	6.08	1.76
	*K_OC_*	290	253	186	1350	510	138
Atenolol	*K_f_*	2.79	29.6	55.1	155	50.0	107
	*n*	1.28	0.55	0.69	0.48	0.61	1.16
	*R* ^2^	0.92	0.99	0.48	1.00	0.94	0.97
	*SE*	2.47	4.28	24.3	2.47	2.37	0.18
	*K_d_*	5.38	10.5	27.1	46.6	20.1	154
	*K_OC_*	465	902	2340	3950	1690	12,100
Caffeine	*K_f_*	2.65	8.63	2.29	15.3	23.0	6.71
	*n*	0.93	0.69	1.07	0.84	0.51	0.69
	*R* ^2^	0.96	0.95	0.93	1.00	0.99	0.99
	*SE*	1.37	3.98	2.05	1.99	3.17	1.76
	*K_d_*	2.23	4.21	2.71	10.5	7.50	3.25
	*K_OC_*	193	362	234	888	629	256

**Table 4:** Pearson correlation coefficient (*r*) and significance level (*p*) of correlations between the SOC content and pH of the soil samples equilibrated with the five model compounds and the resulting linear sorption coefficients (*K_d_*) of the three tested PhACs. Significant correlations are highlighted in bold.

		Sulfadiazine	Atenolol	Caffeine
SOC ^a^	*r*	−0.164	**0.942**	−0.049
	*p*	0.756	**0.005**	0.927
SOC ^b^	*r*	0.518	0.366	0.785
	*p*	0.371	0.544	0.116
pH ^a^	*r*	**−0.918**	−0.181	**−0.827**
	*p*	**0.010**	0.732	**0.042**
pH ^c^	*r*	0.034	−0.501	−0.274
	*p*	0.957	0.390	0.655

^a^ All data; ^b^ data for samples with addition of C:19 excluded; ^c^ data for samples with addition of acetic acid excluded.

## Data Availability

Data will be made available on request.
